# Metabolic patterns in insulin-sensitive male hypogonadism

**DOI:** 10.1038/s41419-018-0588-8

**Published:** 2018-04-22

**Authors:** Giuseppina Fanelli, Federica Gevi, Antonio Belardo, Lello Zolla

**Affiliations:** 1 Department of Ecological and Biological Sciences (DEB), University of Tuscia, Viterbo, Italy; 2 Department of Science and Technology for Agriculture, Forestry, Nature and Energy (DAFNE), University of Tuscia, Viterbo, Italy

## Abstract

Male hypogonadism is a disorder characterised by low levels of the hormone testosterone. At beginning subjects with low levels of testosterone do not show insulin resistance (insulin-sensitive patients), which develops over time (insulin-resistance patients). To analyse the metabolic alterations mainly related to decreased testosterone, we performed metabolomics investigations on the plasma of males with hypogonadism who showed normal insulin levels. Plasma from patients with low testosterone (<8 nmol/l) and homeostatic model assessment for insulin-resistance-index (HOMAi) < 2.5, as well as matched controls, was analysed by UHPLC and mass spectrometry. Then metabolites were then subjected to multivariate statistical analysis and grouped by metabolic pathways. Glycolysis was not altered, as expected for the presence of insulin activity, but imbalances in several other pathways were found, such as the pentose phosphate pathway (PPP), glycerol shuttle, malate shuttle, Krebs cycle (TCA) and lipid metabolism. The PPP was significantly upregulated. Moreover, while the first steps of the Krebs cycle were downregulated, 2-oxoglutarate was replenished via glutaminolysis. Since glutaminolysis leads to an activation of the malate aspartate cycle, greater amounts of NADH and ATP with respect to the control were recorded. The activation of the glycerol shuttle was also recorded, with consequent lower triglyceride production and downregulation of beta-oxidation. This explained the moderately increased dyslipidaemia, as well as the mild increase in body mass index (BMI) observed in insulin-sensitive hypogonadism. Finally, a significant decrease in carnosine was recorded, explaining the muscle weakness commonly observed.

## Introduction

Male hypogonadism is a disorder characterised by low levels of the hormone testosterone^1^. It affects 6–12% of men aged between 40 and 69 years and is strongly associated with metabolic disorders. Diabetes, hypertension, dyslipidaemia and obesity are significantly more frequent in hypogonadic male than eugonadic subjects^2–4^. Testosterone exerts a wide range of beneficial physiological effects critical for men’s health, playing a key role in carbohydrate, fat and protein metabolism. Symptoms associated with the deficiency of this hormone include sexual dysfunction, depressed mood, decreased motivation, fatigue and a lower quality of life^5,6^. Testosterone has a major influence on body fat composition and on maintenance of bone and muscle mass. It has a significant role in glucose homoeostasis and lipid metabolism^7^. Recent evidence suggests that the hypogonadal state actually promotes metabolic alterations through different mechanisms^8–11^. In this regard, a hypogonadal state related to lower testosterone concentration can start in normoinsulinaemic patients (insulin-sensitive (IS) patients) but over the time, blood insulin concentration increases (insulin-resistance (IR) patients), leading to metabolic alterations and clinical complications. In fact, testosterone deficiency is associated with increased fat mass, reduced insulin sensitivity, impaired glucose tolerance, elevated triglycerides (TGs) and free cholesterol but low high-density lipoprotein-cholesterol. IR is associated with an increased risk of testosterone deficiency, and conversely testosterone treatment can improve insulin signal transduction^12^.

Striated muscular, cardiac tissues and adipose tissue are insulin-dependent because they have higher levels of the glucose transporter 4 (GLUT-4) than other tissues. GLUT-4 is normally activated in the presence of insulin and is one of the molecules responsible for glucose entry into cells^13^. Since different metabolic pathways can be altered in hypogonadic males showing normal or high insulin, their identification could help to improve diagnosis, therapy and monitoring of hypogonadism. For these reasons, we performed two parallel investigations, in normoinsulinaemic hypogonadic males and hyperinsulinaemic subjects. The two groups were selected by homeostasis model assessment of IR-index (HOMAi), the most common index to assess insulin sensitivity^14^.

In both investigations, metabolomics analysis of plasma from patients was performed. Metabolomics focuses on the study of low-molecular-weight biochemical molecules (metabolites) in cells, tissues and biofluids. It involves the comprehensive, simultaneous and systematic profiling of many metabolite concentrations and their fluctuations in response to disease, drugs, diet and lifestyle. High-resolution mass spectrometry (HRMS) methods are able to check a huge amount of spectral features in human plasma. Increasing evidence shows that metabolomics could have an impact on diagnosis, prognosis, drug efficacy and safety of several diseases^15^.

The analysis of metabolites was performed in plasma because it is the final collector of molecules produced from all tissues. Testosterone has a complex and different regulatory influence on metabolism of the major tissues involved in insulin action, including liver, adipose tissue and muscle. Moreover, this approach highlights the importance of taking multiple tissues into account and thus taking a systems biology approach.

In this investigation, by performing a broad-spectrum metabolite profile screen in plasma of hypogonadal men showing normal values of insulin (IS patients), testosterone concentration (lower <8 nmol/l), HOMAi < 2.5 and body mass index (BMI; 23.48 ± 3.011), we were able to better identify how low testosterone can affect metabolism pathways, independently of insulin’s contribution or antagonism of insulin signalling. In a parallel investigation^16^, we were studying hypogonadal patients with low testosterone (<8 nmol/) and HOMAi > 2.5 (IR patients).

Interestingly, in the IS hypogonadal males, significant differences were recorded with respect to the control and to IR patients. In IS hypogonadal males, as expected, glycolysis was not altered, probably related to insulin activity, but pathway analysis identified imbalances in several pathways, such as the pentose phosphate pathway (PPP), malate shuttle and Krebs cycle (tricarboxylic acid; TCA). Significantly upregulated glutaminolysis was recorded, which stimulated the right side of the Krebs cycle, suggesting that this cycle is fuelled from glutamine through glutaminolysis. Other lipid pathways were altered.

## Materials and methods

### Patients samples: study design and participants

We evaluated 15 hypogonadal male patients and 15 age- and BMI-matched controls (Table 1). All subjects enroled were informed about the study protocol and gave their written consent. The diagnosis of hypogonadism was based on the presence of clinical symptoms related to this condition (e.g., delayed sexual development, reduced libido or erectile dysfunction) and on the results of standard hormonal exams (total testosterone < 8 nmol/l). The patients affected by hypogonadism were included only if they had HOMAi < 2.5. The participants of the control group were healthy males who were referred to the Outpatient Clinic of Endocrinology and Metabolism for check-up. As shown in Table 1, no differences were found in baseline characteristics between groups.

**Table 1:** Characteristics of study participants

	Ctrl	IS	*p*-value
Subjects (*n*)	15	15	—
Age (years)	42.6±14.41	40.5±158	0.7695720
BMI (kg/m^2^)	23.94±2.54	25.52±3.01	0.2373428
Testosterone	20.87±7.36	6.09±4.49	0.0010053**
Glucose	94±31.05	81±13.09	0.7845517
Insuline	7.06±2.10	6.68±3.03	0.7611147
HOMAi	1.64±0.72	1.35±070	0.3799209
TG (mmol/l)	87.8±45.21	118.4±66.10	0.4524867
Cholesterol (mmol/l)	203.4±34.50	213.6±44.82	0.5842583
HDL cholesterol (mmol/l)	55.8±10.76	53.1±15.73	0.6715782
LDL cholesterol (mmol/l)	129.6±32.14	136.6±40.83	0.6871229

Subjects (*n*)	15	15	
Age (years)	42.6±14.41	49.13±13.5	
BMI (kg/m^2^)	23.94±2.54	30.48±3.011	
Testosterone	20.87±7.36	5.53±3.36	
Glucose	94±31.05	106.13±24.65	
Insuline	7.06±2.10	18.85±6.94	
TG (mmol/l)	87.8±45.21	226±31.2	
Cholesterol (mmol/l)	203.4±34.50	235.13±39.16	
HDL cholesterol (mmol/l)	55.8±10.76	42±15.44	
LDL cholesterol (mmol/l)	129.6±32.14	142.75±34.7	

Data are presented as the mean ± SD. Statistical differences were determined using Tukey’s multiple comparisons where significant interactions were observed

*BMI* body mass index, *TG* triglyceride, *LDL* low-density lipoproteins, *HDL* high-density lipoproteins

**p < 0.01

### Plasma collection and metabolite extraction

Metabolites were extracted by adding 200 µl of each plasma sample to 600 µl of cold (−20 °C) chloroform:methanol:water (1:3:1 ratio). Samples were vortexed for 1 min and left on ice for 2 h for complete protein precipitation. The solutions were then centrifuged for 15 min at 15 000 × *g*.

### Ultra-high-performance liquid chromatography-HRMS

A volume of 20 µl of extracted plasma were injected into an ultra-high-performance liquid chromatography (UHPLC) system (Ultimate 3000, Thermo) and run in positive mode: samples were loaded onto a Reprosil C18 column (2.0 mm × 150 mm, 2.5 μm—Dr Maisch, Germany) for metabolite separation. Chromatographic separations were achieved at a column temperature of 30 °C and flow rate of 0.2 ml/min. For positive ion mode (+) mass spectrometry (MS) analyses, a 0–100% linear gradient of solvent A (ddH2O, 0.1% formic acid) to B (acetonitrile, 0.1% formic acid) was employed over 20 min, returning to 100% A in 2 min and a 6-min post-time solvent A hold. Acetonitrile, formic acid and HPLC-grade water and standards (≥98% chemical purity) were purchased from Sigma Aldrich. The UHPLC system was coupled online with a mass spectrometer Q Exactive (Thermo) scanning in full MS mode (2 μs cans) at 70 000 resolution in the 67–1000 *m*/*z* range, with a target of 1106 ions, maximum ion injection time of 35 ms, 3.8 kV spray voltage, 40 sheath gas and 25 auxiliary gas, operated positive ion mode. Source ionisation parameters were spray voltage, 3.8 kV; capillary temperature, 300 °C; and S-Lens level, 45. Calibration was performed before each analysis against positive- or negative-ion-mode calibration mixes (Piercenet, Thermo Fisher, Rockford, IL) to ensure sub-ppm error of the intact mass. Metabolite assignments were performed using computer software (Maven, 18 Princeton, NJ), upon conversion of raw files into.mzXML format through MassMatrix (Cleveland, OH).

### Metabolomic data processing and statistical analysis

Raw files of replicates were exported, converted into mzXML format through MassMatrix (Cleveland, OH) and then processed by MAVEN software (http://maven.princeton.edu/)^17^. MS chromatograms were elaborated for peak alignment, matching and comparison of parent and fragment ions, and tentative metabolite identification (within a 2 ppm mass-deviation range between observed and expected results against the imported Kyoto Encyclopedia of Genes and Genomes (KEGG) database). To further explore the metabolic differences between the two groups of subjects, multivariate statistical analyses were employed on an MS data set consisting of 15 controls and 15 hypogonadal men. Multivariate statistical analyses were performed on the entire metabolomics data set using the MetaboAnalyst 3.0 software, which also served to overview the data variance structure in an unsupervised manner. Before the analysis, raw data were normalised by median and auto-scaling to increase the importance of low-abundance ions without significant amplification of noise. The web-based tools Metabolite Set Enrichment Analysis (MSEA) and Metabolomic Pathway Analysis (MetPA), which are incorporated into MetaboAnalyst platform, were used to perform metabolite enrichment and pathway analyses, respectively. Data for metabolites detected in all samples were submitted into MSEA and MetPA with annotation based on common chemical names. Accepted metabolites were verified manually using Human Metabolome Database (HMDB), KEGG and PubChem databases. A *Homo sapiens* pathway library was used for pathway analysis. Global test was the selected pathway enrichment analysis method, whereas the node importance measure for topological analysis was the relative betweenness centrality. For MSEA metabolites, data were mapped according to HMDB, and the “metabolite pathway associated metabolites set” library (currently 88 entries) was chosen for the enrichment analysis, which was performed using the package global test. Results were graphed with Graphpad Prism 5.01 (Graphpad SoftwareInc). Statistical analyses were performed with the same software. Data are presented as the means ± SD.

## Results

**Fig. 1:**
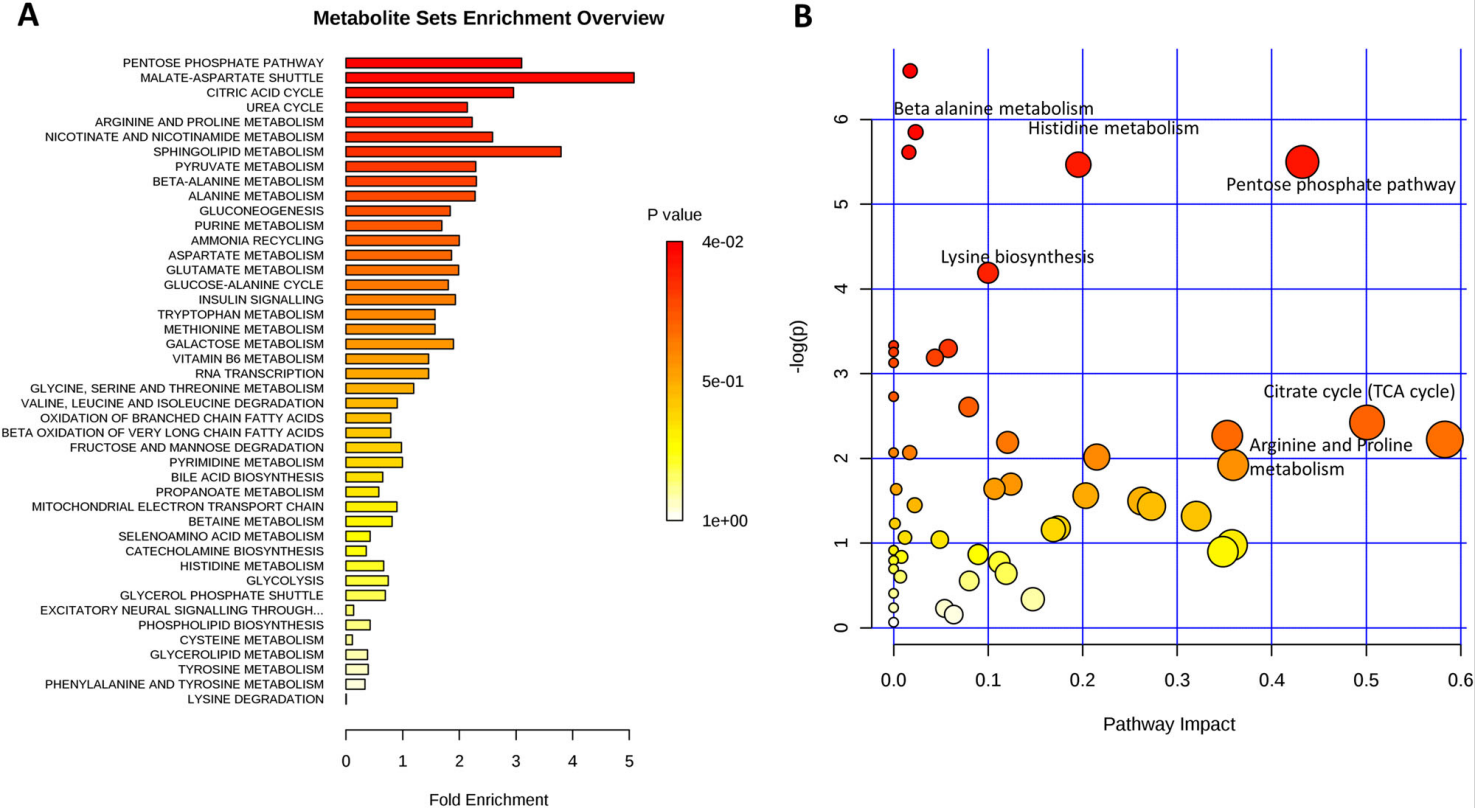
Metabolic Set Enrichment Analysis (MSEA) showing the most altered metabolites revealed in the plasma of hypogonadal men. Colour intensity (white to red) reflects increasing statistical significance, while the circle diameter covaries with pathway impact. **a** The graph was obtained by plotting on the *y*-axis the −log of *p*-values from pathway enrichment analysis and on the *x*-axis the pathway impact values derived from pathway topology analysis. Metabolic Pathway Analysis (MetPA). All the matched pathways are displayed as circles. The colour and size of each circle are based on the *p*-value and pathway impact value, respectively. **b** The graph was obtained by plotting on the *y*-axis the −log of *p*-values from the pathway enrichment analysis and on the *x*-axis the pathway impact values derived from the pathway topology analysis

To explore the metabolic differences in IS male hypogonadism, 15 plasma samples from control subjects and 15 from IS hypogonadal patients were analysed by UHPLC and MS, a highly sensitive, accurate and unbiased approach. To identify which metabolic pathways were most affected in hypogonadal patients, we performed an overview of *p*-values from enrichment analysis and impact values from topology analysis. The “metabolome view”, showing all metabolic pathways, was arranged according to the scores from enrichment analysis (*y*-axis) and from topology analysis (*x*-axis) with the most significant *p*-values in red, the least significant in yellow and white. To understand the biological meaning of the observed metabolic changes, we performed a functional enrichment analysis of the experimental data by MetaboAnalyst 3.0, which performs MSEA for human and mammalian species. The analysis was based on several libraries containing ∼6300 groups of biologically meaningful metabolite sets collected primarily from human studies. Figure 1a shows the results of pathway enrichment analysis conducted by MetaboAnalyst 3.0. From this analysis, the most affected metabolic pathways related to hypogonadism are shown in red, while those altered to a lesser extent are in orange. The “metabolome overview” obtained through MetPA showed the PPP, TCA and β-alanine metabolism were the most affected metabolic pathways in hypogonadic men (Fig. 1b).

**Fig. 2:**
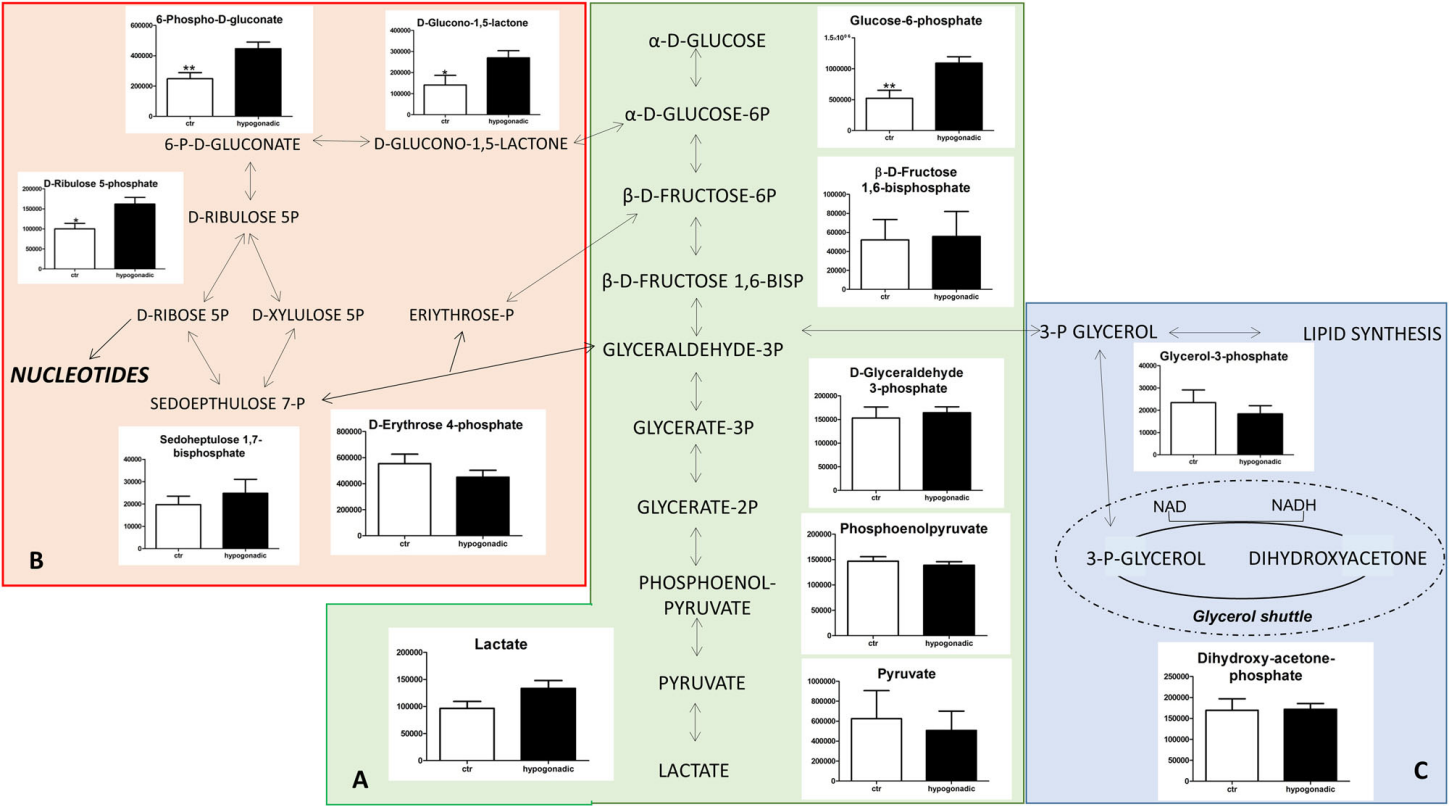
Altereted pathways in hypogonadic IS patients. Intermediates of glycolysis (**a**). Intermediates of the pentose phosphate pathway (**b**). Total amount of PPP metabolites in the plasma appears to be increased with respect to the control. Intermediates of the glycerol shuttle (**c**). The columns represent the mean ± SD (*n* = 15) metabolite concentration over hypogonadal plasma. *p < 0.05, **p < 0.01, against hypogonadal men

Instead glycolysis was not significantly altered (Fig. 2a), indicating that in these patients, glucose was used in muscle, adipose and liver as the main biofuel, and alternative sources were minimally used. Gluconeogenesis was not active. Moreover, as shown in Fig. 2c, 3-glycerol was not significantly used to produce TGs but rather in the glycerol shuttle, since no accumulation of dihydroxyacetone was observed. This leads to the production of NADH, in minor amounts with respect to the control.

**Fig. 3:**
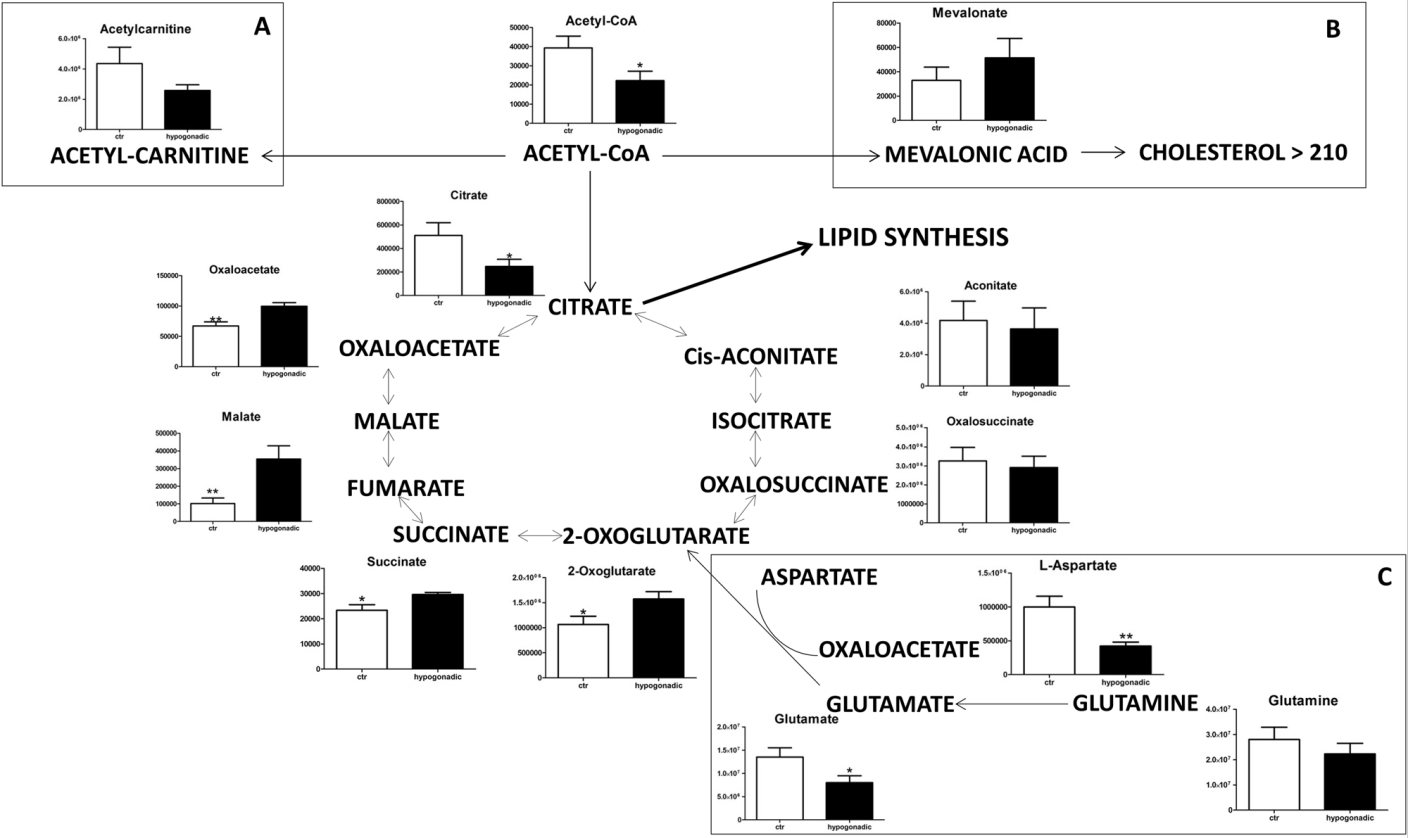
Intermediates of TCA measured in the plasma of hypogonadal patients, revealing that only the first part of this metabolic pathway was reduced. Acyl-carnitine, fundamental to the transport of fatty acids into mitochondria, was less highly produced from acetyl-CoA (**a**). A portion of acetyl-CoA was converted into mevalonic acid and then into cholesterol (**b**). The replenishment of TCA intermediates was due of glutaminolysis (**c**) a stepwise process by which glutamine is converted into glutamate, which is in turn transformed into α-ketoglutarate and aspartate, which are converted to oxaloacetate. Metabolites are expressed as the mean ± SD (*n* = 15) concentration over hypogonadal plasma. *p < 0.05, **p < 0.01, against hypogonadal men

**Fig. 4:**
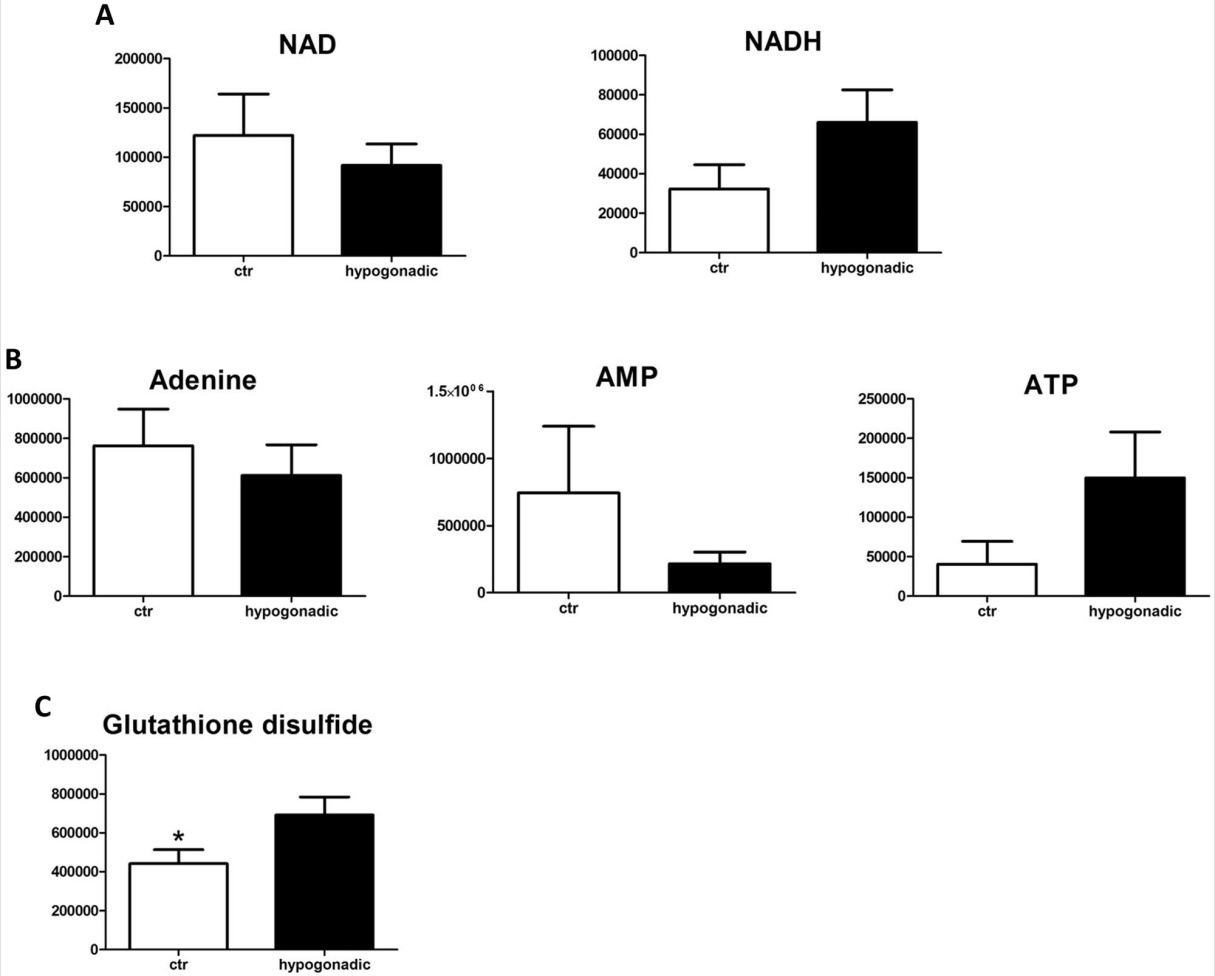
Energy metabolism. The replenishment of TCA intermediates through glutaminolysis leads to activation of the malate aspartate cycle, increased of NADH and ATP, and decreased AMP and NAD (**a**, **b**). Increased glutathione disulphide as a marker of oxidative stress (**c**). Metabolites are expressed as the mean ± SD (*n* = 15) concentration over hypogonadal plasma. *p < 0.05, against hypogonadal men

Interestingly, IS hypogonadism is associated with significant lactate production. The PPP was strongly upregulated (Fig. 2b), indicating an oxidative stress, as confirmed by the increased Oxidized glutathione (GS-SG) accumulation (Fig. 4c). Pyruvate concentration was low, while possible precursors of pyruvate, such as cysteine, tyrosine and alanine, increased significantly in plasma ruling out their involvement in pyruvate production.

The level of acetyl-CoA was slightly reduced (Fig. 3) respect to the control, indicating a deficiency in terms of energy production, but within the limits of acceptability. Acyl-carnitine, essential for the import of fatty acids into mitochondria, was produced from acetyl-CoA to a lesser extent compared to controls (Fig. 3a), indicating a reduced β-oxidation of fatty acids. A small portion of acetyl-CoA is converted into mevalonic acid (Fig. 3b) and then into cholesterol, which is increased in IS hypogonadal males to 235.13 ± 39.16 (Table 1).

A significant amount of acetyl-CoA entered the Krebs cycle, although less than in controls, as indicated by the decreased citrate, succinate and aconitate levels. Interestingly, a restoration of the Krebs cycle was observed starting from oxoglutarate. This replenishment of TCA intermediates was due to glutaminolysis, a process by which glutamine is converted into glutamate, which is in turn transformed into α-ketoglutarate, and finally into aspartate and thence to oxaloacetate (Fig. 3c). The consequent increase in malate leads to an activation of the malate aspartate cycle, with an increase in NADH and ATP, and a decrease in AMP and NAD (Fig. 4a, b).

**Fig. 5:**
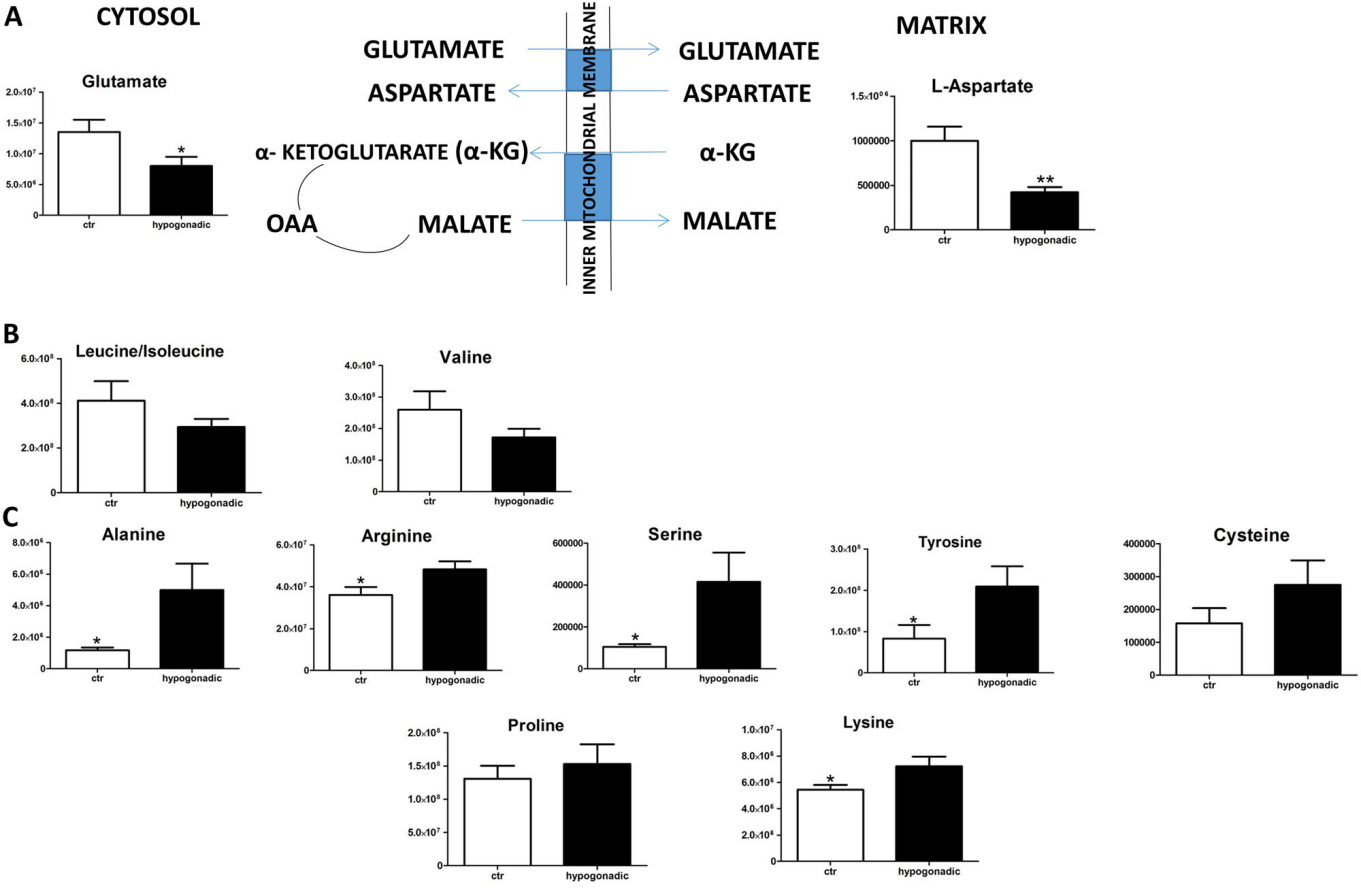
Amino acids metabolism. Amino acids involved in the malate-aspartate shuttle (**a**). Decreases branched-chain amino acids (BCAA) leucine, isoleucine and valine (**b**). Plasma amino acids that were significantly increased (**c**). Metabolites are expressed as the mean ± SD (*n* = 15) concentration over hypogonadal plasma. *p < 0.05, **p < 0.01, against hypogonadal men

**Fig. 6:**
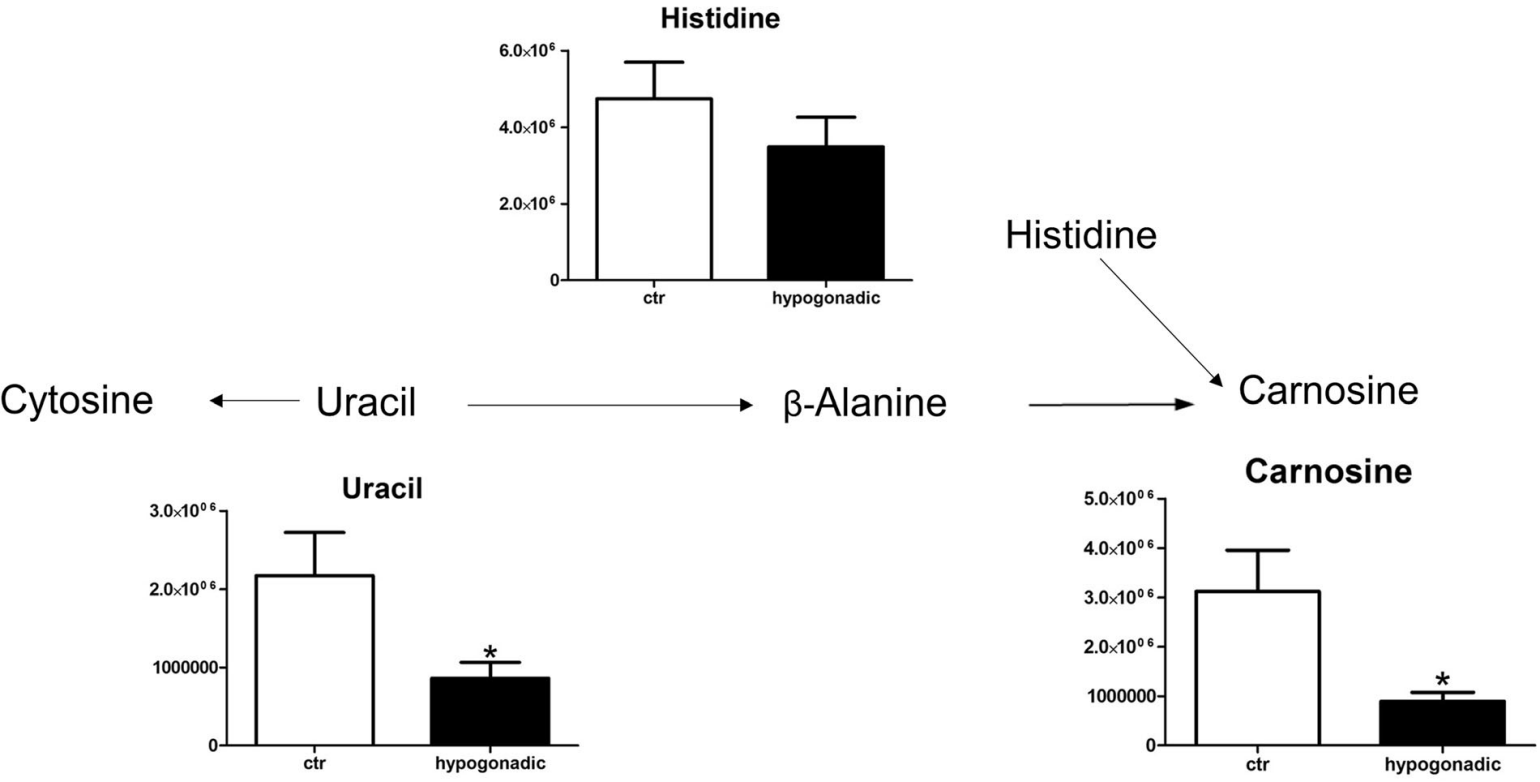
Metabolism of carnosine production from β-alanine. The columns represent mean ± SD (*n* = 15) concentration over hypogonadal plasma. *p < 0.05, against hypogonadal men

In IS hypogonadism subjects, most amino acids did not undergo strong alterations (Supplementary Figure 1), except glutamine and glutamate as a consequence of the upregulated malate shuttle (Fig. 5a). It is of note a slight decrease of branched-chain amino acids, such as valine, leucine and isoleucine, (Fig. 5b), but to a lesser extent than observed in IR male hypogonadism^16^. An increase of proline and lysine suggests a lower synthesis of collagen fibres (Fig. 5c). Finally, carnosine, as well as its precursors β-alanine and uracil, is strongly decreased (Fig. 6), indicating that insulin does not influence this metabolism.

## Discussion

Our HRMS metabolomics analysis revealed that several canonical biochemical pathways were altered in IS male hypogonadism respect to the controls. In these patients, being insulin still active, all the changes should be only related to the decreased testosterone concentration. Once compared with the analysis performed in IR male hypogonadism, these metabolite changes will allow us to better understand the role played by insulin when patients become insulin-resistant over time. Thus, the present observations in the first stage of hypogonadism, when insulin levels are mostly normal, may have an impact not only on diagnosis and prognosis but also on drug treatment efficacy and safety.

As expected, glycolysis is not altered in IS male hypogonadism, since insulin is still operating thus allowing tissues to use glucose as a main fuel. Therefore, it is not surprising that in the plasma of hypogonadal men, the glycaemia was similar to controls. Consequently, gluconeogenesis is not strictly required to produce endogenous glucose, at variance with IR subjects^16^. This claim is in agreement with Martin et al.^18^, who showed that the expression of several genes in glycolysis, glycogen metabolism, TCA cycle and PPPs was significantly increased by androgen deprivation therapy (ADT). The PPP was activated, as found by Kelly et al.^19^, who showed that in patients submitted to ADT for prostate cancer, the mRNA expression of glucose-6-phosphate dehydrogenase (G6PD) was elevated. In fact, G6PD is the gateway enzyme of the PPP in the liver of mice that display very low testosterone and non-functional androgen receptors. Activation of the PPP pathways allows the cells to produce NADPH as an alternative energy source. An increase in GS-SG confirmed that these patients showed oxidative stress, in agreement with Haymana et al.^20^.

Surprisingly, lactate was high in IS hypogonadal males, at variance with IR hypogonadism^16^. Consequently, in view of the correlation between lactate and testosterone production in rat Leydig cells^21^, in these patients the testosterone production seems to be still stimulated^22^.

Interestingly, in IS hypogonadism, 3-phosphoglycerolphosphate was prevalent in the glycerol shuttle, and it was not diverted to produce TGs, in contrast to IR hypogonadism^16^. In this stage of hypogonadism, the liver is oriented to lipogenesis, increasing the amount of TGs with respect to the controls, even though a lesser extent than in IR (118.4 mmol/l in IS and 226 mmol/l in IR). Consequently, the BMI increased (BMI ranged 25.52 kg/m^2^) respect to the control (22.02 kg/m^2^) but less whether compared to IR hypogonadism (30.48 kg/m^2^)^16^. Acyl-carnitine production was decreased in IS hypogonadism (Fig. 3a) indicating a lower burning of fatty acids by β-oxidation^23^.

A variation of the lipid composition (both in terms of lipid types and in terms of fatty acid composition) between IS and IR has already been observed in our laboratory and is still under investigation. Cholesterol production slightly increased in IS hypogonadism, as did its precursor mevalonic acid (but not significantly), in agreement with the hypothesis that low testosterone promotes cholesterol mobilisation from the liver^24^. Surprisingly, our metabolomics analysis revealed that the Krebs cycle was stopped at the citrate-isocitrate level, but the cycle restarted at the level of 2-oxoglutarate. This was due to the glutaminolysis process, where glutamine is converted into glutamate and finally into 2-oxoglutarate, which is activated in IS male hypogonadism, and not in IR hypogonadism^16^. This process is upregulated in tumour cells, and represents the main source of energy in cancer cells^25^. A relationship between insulin secretion and glutamate dehydrogenase was also observed in pancreatic cells in type 2 diabetes^26^. In this case, the release of insulin is increased by activation of glutaminolysis, in agreement with data observed in our patients, which suggested that their pancreatic β-cells were trying to produce more insulin. Our results are in agreement with those reported by Adams et al.^23^, who detected potential links between the TCA cycle, fatty acids metabolism, acyl-carnitine and IR. Thus, the activation of glutaminolysis represents an adaptive reaction of cells to produce energy as a consequence of the deficiency of testosterone but only when insulin is active, while it disappears in the IR stage^16^. This hypothesis deserves further investigation to allow therapeutic intervention when hypogonadism is at the first stadium. No significant changes were observed in plasma amino acids in IS hypogonadism in agreement with Mauras et al.^27^, except for aspartate and glutamate, which in fact decreased due to the glutaminolysis. It is of note that proline and lysine increase in hypogonadism. Since these two amino acids participate in collagen fibre formation, it may be hypothesised that their accumulation in plasma is an indication of slower bone formation. This could explain the osteoporosis commonly observed in hypogonadism^28,29^ and related to a decrease in testosterone^30,31^ independent of insulin activity. Finally, degradation of uracil produces β-alanine (Fig. 6), the precursor of carnosine. All of these three metabolites significantly decreased in hypogonadism, suggesting that this process is controlled by testosterone. This hypothesis is in line with Penafiel et al.^32^ results, showing a reestablishment of carnosine levels after testosterone replacement. β-Alanine supplementation can significantly increase intramuscular carnosine, which then may improve exercise performance^33^. According to Varanoske et al.^34^, high intramuscular carnosine may attenuate fatigue during isokinetic and isometric exercise in recreationally trained athletes.

In conclusion, the analysis of all plasma metabolites and the related altered pathways in male IS hypogonadism patients allowed us to better discriminate the effects of testosterone deficiency, which could not be singled out when the disease evolves into IR. This investigation, when compared with data recorded from IR patents, will help improve diagnosis, therapy and monitoring of hypogonadism and offer a variety of scientific opportunities to improve the clinical management of testosterone deficiency in men.

At this transitional phase of hypogonadism, fewer pathways are altered than in the late stage. Glucose metabolism is only slightly downregulated, with activation of the PPP. The Krebs cycle is downregulated but reactivated at level of 2-oxoglutarate through glutaminolysis activation. The malate shuttle is activated, with higher production of NADH and ATP with respect to the controls.

The glycerol shuttle is still active, but part of the glycerol is used for TG production, although to a lesser extent than in IR. Lipogenesis is slightly activated, whereas β-fatty acid oxidation is slowed. Adiposity is increased but not significantly. Amino acids are not employed for energy production, although glutamine and aspartate are used for glutaminolysis more than in controls. Moreover, a lower metabolism of proline and lysine was recorded, probably related to a decreased collagen production and explaining the lower lean body mass observed in hypogonadism, which, with a lower carnosine formation, contributes to increased fatigue during isokinetic and isometric exercise.

Thus, metabolomics studies appear to be very important for the development of personalised therapy also in the case of hypogonadism.

## Disclaimer

There were no extra sources supporting this research (excluding sources already declared). The study is original, and the manuscript has not been published yet and is not being considered for publication elsewhere in any language, either integrally or partially, except as an abstract. All authors have agreed with the submission in its present (and subsequent) form.

## Electronic supplementary material

Supplemental Figure 1

Supplementary figure legends
