# Supplementary figures and images for: Metabolic patterns in insulin-sensitive male hypogonadism
Source: Cell Death Dis. 2018 Apr 22;9(6):653. doi: 10.1038/s41419-018-0588-8 (PMC5974275; doi:10.1038/s41419-018-0588-8)

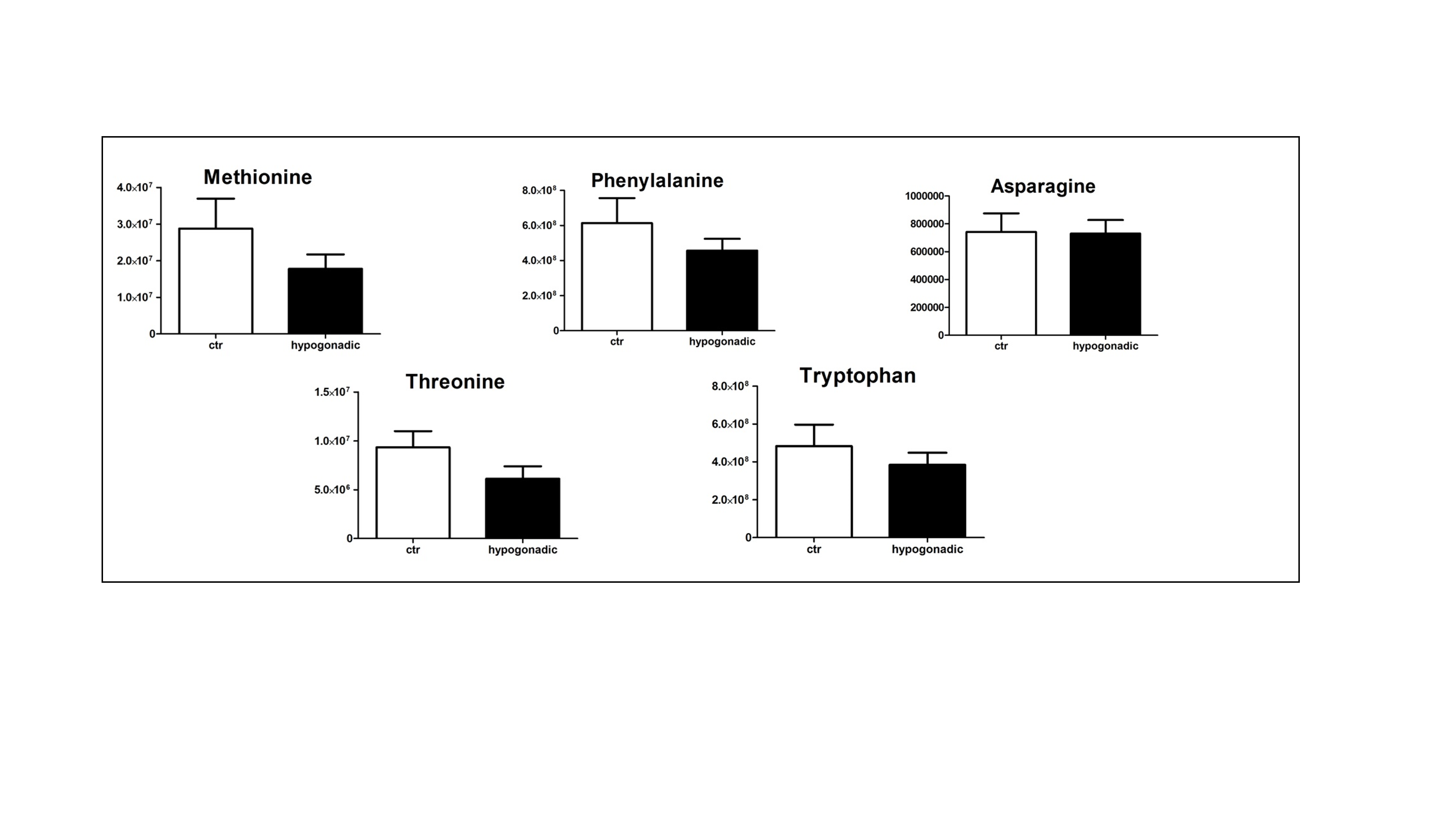

Supplement: Supplementary file 1 — Supplemental Figure 1 [file 41419_2018_588_MOESM1_ESM.tif]
