# Supplementary material for: Metabolic patterns in insulin-sensitive male hypogonadism
Source: Cell Death Dis. 2018 Apr 22;9(6):653. doi: 10.1038/s41419-018-0588-8 (PMC5974275; doi:10.1038/s41419-018-0588-8)
Supplement: Supplementary file 2 — Supplementary figure legends [file 41419_2018_588_MOESM2_ESM.docx]

**Metabolic patterns in insulin-sensitive male hypogonadism.**

**Fanelli Giuseppina, Gevi Federica, Belardo Antonio and Zolla Lello***

**Supplemental Figure 1**: Amino acids that did not show strong alterations.
